# Evaluation of hippocampus-sparing VMAT versus conventional VMAT in locally advanced nasopharyngeal carcinoma: a dosimetric and biological analysis

**DOI:** 10.3389/fonc.2025.1628590

**Published:** 2025-10-08

**Authors:** Zhi Wei Liu, Zhang Rui Liang, Wei Wei Wu, Lei Tan, Song Huan Lin

**Affiliations:** ^1^ Center of Radiation Oncology, Ganzhou Cancer Hospital, Ganzhou, Jiangxi, China; ^2^ Department of Radiology, The First Affiliated Hospital of Xi’an Jiaotong University, Xi’an, Shaanxi, China

**Keywords:** nasopharyngeal carcinoma, volumetric modulated arc therapy, hippocampus-sparing volumetric modulated arc therapy, EUD, NTCP

## Abstract

**Background and purpose:**

Radiation-induced hippocampal damage poses a risk of cognitive decline following radiotherapy. Existing international guidelines for nasopharyngeal carcinoma (NPC) radiotherapy provide insufficient specific guidance on hippocampus sparing, which is critically needed, particularly for patients with locally advanced disease. This study was conducted to retrospectively evaluate the dosimetric feasibility and safety of a hippocampus-sparing VMAT technique for locally advanced NPC. Specifically, we assessed its ability to reduce hippocampal dose while ensuring target coverage and respecting dose limits for other organs at risk based on a comparative analysis of treatment plans.

**Materials and methods:**

This retrospective study included the clinical data and treatment plans of 60 patients previously treated for locally advanced nasopharyngeal carcinoma. For all patients, target volumes and organs at risk (OARs) had been delineated according to international guidelines. Notably, for the purpose of this analysis, the left and right hippocampi were meticulously delineated as independent OARs. For this dosimetric comparison, the cohort was divided into two groups: the conventional group (n=30), consisting of patients treated with a conventional VMAT plan, and the hippocampus-sparing group (n=30), for whom HS-VMAT plans were retrospectively analyzed or generated. A systematic comparative analysis of dosimetric and biological data was subsequently conducted between the two groups.

**Results:**

Consistent with previous studies, hippocampus-sparing plans showed statistically significant differences from conventional VMAT plans in multiple parameters, resulting in significantly reduced radiation doses to both the left and right hippocampi. For instance, left hippocampal Dmax was 35.40 Gy compared to 51.15 Gy in conventional plans (a decrease of 15.75 Gy), and right hippocampal Dmax was 36.47 Gy compared to 47.27 Gy (a decrease of 10.8 Gy). Similar significant reductions were observed for Dmean (left: 11.69 Gy vs 16.52 Gy; right: 11.79 Gy vs 16.00 Gy) and D40% (left: 11.38 ± 6.76 Gy vs 16.42 ± 6.99 Gy; right: 11.50 Gy vs 16.62 Gy). However, the Dmin for the left and right hippocampi did not show a significant decrease. Additionally, slight differences in target homogeneity were noted. EUD and NTCP analyses from both groups further demonstrated that reducing hippocampal dose with the hippocampus-sparing plan effectively lowered the predicted probability of complications in the left and right hippocampal tissue.

**Conclusion:**

This retrospective dosimetric comparison demonstrates the feasibility of the hippocampus-sparing technique in patients with locally advanced nasopharmenyngeal carcinoma. Our analysis indicates that HS-VMAT plans can achieve significant reductions in key hippocampal dose parameters, such as Dmax, Dmean, and D40%, while simultaneously maintaining adequate target coverage and adhering to dose constraints for other organs at risk. Supported by both dosimetric and biological (NTCP) analyses, these findings suggest a significantly lower predicted probability of hippocampus-related complications with the HS-VMAT approach. Therefore, our results support HS-VMAT as a theoretically feasible and clinically valuable strategy, with potential significance for preserving the neurocognitive function of patients with locally advanced NPC.

## 1 Introduction

Nasopharyngeal carcinoma (NPC), a common malignant tumor in the head and neck region, is primarily treated with radiotherapy for patients with locoregionally advanced disease (1, 2). Currently employed radiotherapy techniques, including Intensity-Modulated Radiotherapy (IMRT), Volumetric Modulated Arc Therapy (VMAT), and Tomotherapy, while achieving precise tumor irradiation, inevitably traverse adjacent normal tissues, leading to radiation-induced injury. The hippocampal structure, due to its proximity to the nasopharyngeal target volume, is highly susceptible to high-dose radiation exposure (3–5). Studies have confirmed that radiation-induced hippocampal injury is closely associated with post-radiotherapy sequelae such as language dysfunction, memory decline, and neurocognitive impairment in patients (6–8). Given the relatively high cure rates and long survival duration of NPC patients, the importance of radiotherapy-related late toxicities, particularly the long-term impact on neurocognitive function on patient quality of life, is increasingly recognized. Therefore, how to effectively reduce radiation dose to organs at risk (OARs) adjacent to the target volume in NPC radiotherapy while ensuring adequate target coverage has become a focal point of attention for both domestic and international scholars, with protection of the hippocampus being particularly crucial. Although studies have indicated that implementing hippocampus-sparing techniques in NPC radiotherapy can reduce hippocampal dose without significantly compromising target volume coverage or dose constraints for other OARs, there is still no internationally established dose constraint standard specifically for hippocampal sparing in NPC radiotherapy. Consequently, clinical practice often refers to standards developed for whole-brain radiotherapy. Furthermore, tumor stage, particularly in advanced cases (T3/T4), can lead to the hippocampus receiving higher radiation doses, and the potential issue of hippocampal structure damage therefore warrants urgent attention.

Therefore, this study was conducted to retrospectively analyze the dosimetric and biological impact of a hippocampus-sparing technique in patients with locally advanced NPC. We included 60 patients with T3 or T4 NPC who had previously received VMAT at our institution. These patients were divided into two groups for this dosimetric comparison: one group (n=30) had received Conventional VMAT, and for the other group (n=30), Hippocampus-Sparing VMAT (HS-VMAT) plans were retrospectively generated or analyzed. This study focused on comparing the radiotherapy plans of the two groups, evaluating the dose coverage of target volumes, and assessing whether the received doses to key OARs met clinical constraints. More importantly, we precisely calculated and compared the dosimetric parameters of the hippocampal structure in both groups (following the approach by Tsai PF to analyze the left and right hippocampi as independent OARs). To quantitatively assess the potential of HS-VMAT in reducing the risk of hippocampal injury, we calculated and compared the normal tissue complication probability (NTCP) for the hippocampus in both groups based on the dosimetric data.

Through these retrospective dosimetric and biological analyses, this study aimed to determine whether HS-VMAT could have significantly reduced hippocampal dose and potential injury risk while ensuring sufficient tumor coverage and meeting dose constraints for other OARs. This analysis provides important evidence for establishing future hippocampal dose constraint standards for NPC radiotherapy and promoting safer and more effective radiotherapy techniques.

## 2 Materials and methods

### 2.1 Patient selection and image acquisition

This study retrospectively included a total of 60 patients with locoregionally advanced T3 or T4 nasopharyngeal carcinoma who received radiotherapy at Ganzhou Cancer Hospital from January 2023 to August 2024. Prior to radiotherapy planning, all patients underwent Computed Tomography (CT) simulation with contrast enhancement of the head and neck region. The scanning range extended from approximately 2 cm superior to the eyebrows to approximately 3 cm inferior to the clavicular heads, using a slice thickness of 3 mm for continuous axial acquisition. To ensure accurate patient positioning and reproducibility for radiotherapy, patients were positioned using a surface laser positioning system before scanning, and external fiducial markers were placed on their individualized head masks. Following the CT scan, all patients further underwent magnetic resonance imaging (MRI) scans to aid in the precise delineation of tumor volumes and organs at risk. Before the MRI scan, the external fiducial markers on the head masks were carefully verified to ensure consistent patient positioning with that of the CT scan. A head and neck coil was used for scanning, with a scanning range extending from the frontal sinus down to 3 cm below the clavicular heads. Acquired sequences included Spin Echo (SE) T1-weighted (T1W), T2-weighted (T2W), and Fluid-Attenuated Inversion Recovery (FLAIR) sequences. Given that T1W sequences exhibit high signal-to-noise ratio and good contrast, they were primarily used for subsequent image registration and delineation. All scanning sequences had a slice thickness of 3 mm. All methods in this study were carried out in accordance with the relevant guidelines and regulations of Helsinki Declaration, All the experimental protocols have been approved by the Medical Ethics Committee of Ganzhou Cancer Hospital, the need for informed consent to participate was waived by the Medical Ethics Committee of Ganzhou Cancer Hospital.

### 2.2 Target volume and OAR delineation

For each patient’s radiotherapy plan, CT and MR images were co-registered to leverage the superior soft-tissue contrast of MRI for accurate contouring. The delineation of all target volumes and OARs was independently performed by two experienced radiation oncologists and subsequently cross-checked to ensure consistency and accuracy. The tumor volumes were delineated in accordance with standard clinical guidelines. The Gross Tumor Volume (GTV) included the primary nasopharyngeal tumor (GTVnx) and any metastatic cervical lymph nodes (GTVnd), as identified on clinical and imaging examinations. The high-risk Clinical Target Volume (CTV1) was defined as the GTVnx with a margin to account for potential subclinical extension, while the low-risk CTV (CTV2) encompassed other relevant high-risk lymphatic regions. Planning Target Volumes (PTVs) were generated by applying a 3-mm isotropic expansion to the corresponding CTVs (PTV1 and PTV2). Major OARs, including the brainstem, spinal cord, optic chiasm, and parotid glands, were initially contoured using an auto-segmentation tool within the United Imaging Healthcare planning system and were subsequently reviewed and manually edited by the radiation oncologists as necessary. A key methodological aspect of this study was the meticulous, manual delineation of the left and right hippocampi as two independent OARs. This was performed on the fused CT-MR images, following the contouring atlas by Tsai PF et al. (9), to provide a robust basis for the detailed, bilateral dosimetric and biological analyses central to our investigation.

### 2.3 Treatment planning

All 60 patients included in this retrospective study had been prescribed a total dose of 70 Gy in 32 fractions, delivered via a Varian VitalBeam linear accelerator. For this dosimetric comparative analysis, the cohort was divided into two groups based on the treatment planning strategy. The Conventional Group (n=30) consisted of patients whose original treatment was delivered using a standard conventional VMAT plan. For the Hippocampus-Sparing (HS) Group (n=30), HS-VMAT plans were retrospectively generated for the purpose of this study, using the same CT datasets and delineated structures. All plans, both original and retrospectively generated, were designed on the Eclipse planning system (v15.6.8) using a three-full-arc VMAT technique. The optimization process for all plans was standardized to ensure strict adherence to target volume coverage criteria and dose constraints for critical OARs, as detailed in **Table 1**. The crucial difference in the optimization process for the HS-VMAT plans was the addition of specific dose constraints for the hippocampal structures. Guided by the dose thresholds from the NRG Oncology CC001 trial, the optimization objectives for the hippocampus were set as: Dmax ≤ 17 Gy and Dmean ≤ 9 Gy. This was aimed at minimizing the hippocampal dose as much as achievable while maintaining the primary objectives for target coverage and the sparing of other critical organs.

**Table 1:** Dose limits for OARs.

OARs	Constraints (desirable dose)	OARs	Constraints (desirable dose)
Brainstem	Dmax ≤ 60Gy	Lens	Dmax ≤ 8Gy
Spinal cord	Dmax ≤ 50Gy	Temporal lobe	Dmax ≤ 70Gy
Optic chiasm	Dmax ≤ 60Gy	Parotid gland	V30Gy ≤ 50%
Optic nerve	Dmax ≤ 60Gy	Eyes	Dmean ≤ 30 Gy

### 2.4 Statistical analysis

All statistical analyses were performed using SPSS 26.0 software. Descriptive statistics were used to summarize patient and dosimetric data. Categorical variables, such as clinical baseline characteristics, were presented as counts and percentages (n, %) and compared using the Chi-square (χ²) test. For all continuous variables, the Shapiro-Wilk test was first utilized to assess the normality of their distribution. Normally distributed data were expressed as mean ± standard deviation (SD) and compared between the two groups using the independent samples t-test. Data that were not normally distributed were presented as median and interquartile range (IQR) and compared using the non-parametric Mann-Whitney U test. This methodology was applied to all dosimetric parameters for target volumes and organs at risk. All statistical tests were two-sided, and a P-value < 0.05 was considered to indicate statistical significance.

### 2.5 TPS plan parameter analysis

Various parameters were compared between the two VMAT plan groups to evaluate the dosimetric characteristics of target volumes and OARs. Specifically, we compared the Homogeneity Index (HI) (Equation 1) and Conformity Index (CI) (Equation 2) for the target volumes, and the Dmax, Dmean, Dmin, and D40% values for the hippocampus. All relevant parameters were exported from the treatment planning system (TPS).


(1) $$\begin{array}{c} HI=\frac{D_{2\%}-D_{98\%}}{D_{50\%}} \end{array}$$

(2) $$\begin{array}{c} CI=\frac{V_{D95}}{V_{PTV}} \end{array}$$

The analyzed dosimetric parameters for target volumes typically include:

- D_2_%: The dose received by 2% of the target volume (often representing the maximum dose).
- D_98_%: The dose received by 98% of the target volume (often representing the minimum dose).
- D_50_%: The dose received by 50% of the target volume (often representing the median dose).
- V_D_95_%: The volume receiving at least 95% of the prescribed dose, typically referring to the volume within the target where the dose is no less than 95% of the target dose objective.
- V_PTV: The volume of the Planning Target Volume.

### 2.6 Normal tissue complication probability model

Normal Tissue Complication Probability is an indicator used in radiotherapy to estimate the likelihood of normal tissues developing complications or adverse reactions due to radiation exposure. NTCP is a crucial parameter in treatment planning, aiding in quantifying the risk faced by surrounding healthy tissues after tumor irradiation. It is typically modeled using mathematical formulas based on the normal tissue dose distribution. These models consider factors such as the volume of irradiated tissue, the total dose, and the tissue’s sensitivity to radiation. Commonly used models include the Lyman-Kutcher-Burman (LKB) (10) model, the Logit (11) model, the Biological Effect of Radiation (BEER) model, and some modified versions (12). In this study, we adopted a simplified calculation method for NTCP based on an improved LKB model, as proposed by Cao (13). This simplified formula enhances the practicality and accessibility of NTCP calculation, allowing for direct calculation using a calculator, thereby making NTCP computation more convenient and straightforward. As shown in Equation 5.


(3) EUD=[∑i=1(vi(EQD)2ia)]1a

(4) $$\begin{array}{c} x=\frac{\left(EUD-TD_{50}\right)}{\left(m\times TD_{50}\right)} \end{array}$$

(5) $$\begin{array}{c} NTCP=\varphi (x)=\frac{1}{1+e^{-1.597x-0.071x^{3}}} \end{array}$$

In Equation 3, EUD refers to Equivalent Uniform Dose. Where ‘m’ in Equation 4 reflects the steepness of the dose-response curve, representing the rate at which the risk of damage increases with dose; a lower ‘m’ value indicates a steeper dose-response curve. TD_50_ represents the dose at which there is a 50% probability of complication occurrence. Based on relevant literature (14, 15), we found that for hippocampal tissue, m = 0.15 and TD_50_ = 48 Gy.

## 3 Results

As shown in **Table 2**, there were no statistically significant differences in the relevant clinical baseline characteristics between the two groups.

**Table 2:** Baseline characteristics of two groups of patients with locally advanced nasopharyngeal carcinoma.

Clinical baseline characteristics	Conventional group	Hippocampus-sparing group	T/Z/x^²^ value	p-value
Age	51.867 ± 10.514	49.333 ± 11.545	-0.889	0.378
Sex				
Male	19 (63.3%)	22 (73.3%)	0.693^a^	0.405
Female	11 (36,7%)	8 (26.7%)		
TNM Stage				
T3	20 (66.7%)	23 (76.7%)	0.523^a^	0.390
T4	10 (33.3%)	7 (23.3%)		

Data are expressed as number (percentage) or mean ± standard deviation. Statistical comparisons were performed using the independent samples t-test for age (t-value reported) and the Chi-square test for categorical data (χ² value reported). A Mann-Whitney U test (Z-value reported) would be used for non-normally distributed continuous data. The superscript ‘a’ indicates that the value is a Chi-square (χ²) statistic.

The volumes of the left and right hippocampus were comparable between the two groups, with no statistically significant differences found (**Table 3**). This establishes the comparability of the groups regarding baseline hippocampal volume, allowing for subsequent data comparison. The results presented in **Table 3** from our experiment are consistent with previous studies. The hippocampus-sparing plans showed statistically significant differences from conventional VMAT plans across multiple parameters. Specifically, the radiation doses received by both the left and right hippocampi were significantly reduced in the hippocampus-sparing plans:

**Table 3:** Comparison of metrological parameters of the left and right hippocampus in two groups of patients with locally advanced nasopharyngeal carcinoma under different radiotherapy plans.

Structure	Conventional group	Hippocampus-sparing group	T/Z value	p-value
Left hippocampal Dmax	51.149 ± 14.692	35.401 ± 16.169	-3.983	**<0.001**
Right hippocampal Dmax	47.267 ± 13.248	36.470 ± 15.947	-2.859	**0.006**
Left hippocampal Dmean	16.520 ± 5.862	11.688 ± 6.373	-3.084	**0.003**
Right hippocampal Dmean	16.002 ± 7.031	11.786 ± 6.584	-2.415	**0.019**
Left hippocampal Dmin	3.949 ± 2.645	4.610 ± 3.715	0.802	0.426
Right hippocampal Dmin	3.731 ± 2.402	3.853 ± 1.689	0.229	0.82
Left hippocampal D40%	16.420 ± 6.986	11.384 ± 6.764	-2.880	**0.006**
Right hippocampal D40%	16.624 ± 8.561	11.500 ± 7.682	-2.458	**0.017**
Left hippocampal volume (cm³)	2.730 ± 0.624	2,720 ± 0.614	0.911	0.911
Right hippocampal volume (cm³)	2.967 ± 0.716	2.987 ± 0.662	0.683	0.683

Data are expressed as mean ± standard deviation.

P-values < 0.05 are considered statistically significant and are highlighted in bold.

- Hippocampal Dmax: Left hippocampus decreased by 15.75 Gy (35.40 Gy vs. 51.15 Gy in conventional plans); Right hippocampus decreased by 10.8 Gy (36.47 Gy vs. 47.27 Gy in conventional plans).
- Hippocampal Dmean: Left hippocampus decreased by 4.83 Gy (11.69 Gy vs. 16.52 Gy in conventional plans); Right hippocampus decreased by 4.21 Gy (11.79 Gy vs. 16.00 Gy in conventional plans).
- Hippocampal D_40_%: Left hippocampus decreased by 5.04 Gy (11.38 ± 6.76 Gy vs. 16.42 ± 6.99 Gy in conventional plans); Right hippocampus decreased by 5.12 Gy (11.50 Gy vs. 16.62 Gy in conventional plans).

However, the Dmin for the left and right hippocampi did not show a statistically significant decrease in the hippocampus-sparing plans compared to conventional plans. From **Table 4**, it can be seen that while the dose metrics for the hippocampus were significantly reduced, the uniformity and conformity of the target volumes were largely preserved. The HI and CI for PGTV showed no significant difference compared to conventional VMAT. Similarly, the CI of PTV2 was comparable between the two groups. However, a slight difference in the HI of PTV2 was observed.

**Table 4:** Comparison of target homogeneity, conformity, and OAR dose in two plan groups.

Structure	Conventional group	Hippocampus-sparing group	T/Z value	p-value
PGTV-HI	0.051 ± 0.019	0.048 ± 0.013	-0.636	0.527
PGTV-CI	0.501 ± 0.207	0.447 ± 0.156	-1.159	0.251
PTV2-HI	0.287 ± 0.013	0.293 ± 0.011	2.245	**0.029**
PTV2-CI	0.863 ± 0.024	0.865 ± 0.022	0.461	0.646
Lens-L max	6.567 ± 1.024	6.700 ± 0.873	0.547	0.586
Lens-R max	6.525 ± 1.016	6.708 ± 0.837	0.767	0.446
Spinal cord max	34.722 ± 2.450	33.989 ± 2.232	-1.220	0.227
Brainstem max	58.211 ± 5.334	56.685 ± 5.891	-1.061	0.293

Data are expressed as mean ± standard deviation.

P-values < 0.05 are considered statistically significant and are highlighted in bold.

**Figure 1:**
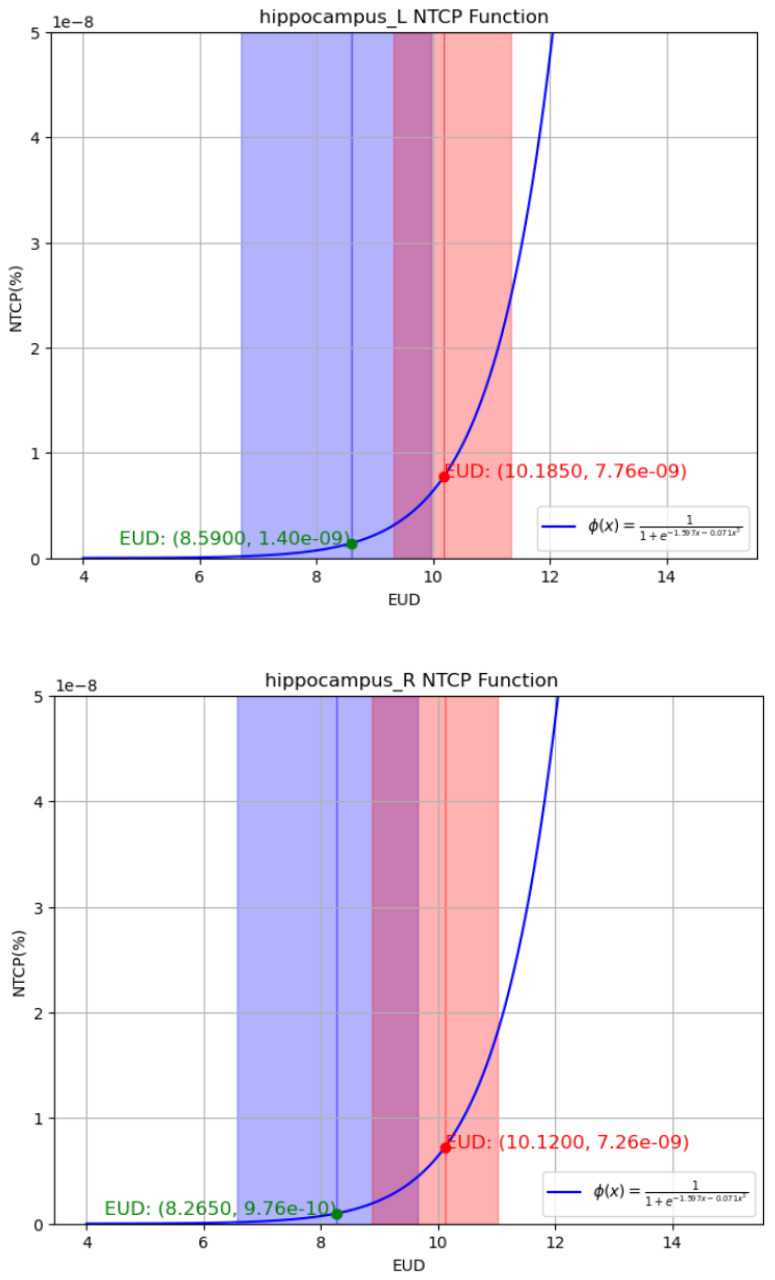
**(A, B)** represent the functional relationship curves of EUD and BTCP of the left and right seahorses. Red represents the EUD and NTCP values of the conventional plan, and blue represents the value of the seahorse preservation plan.

Based on the NTCP model, we calculated the EUD and NTCP values for two groups of data, as shown in **Table 5**. It can be seen that the EUD values differed significantly between the hippocampus-sparing group and the conventional group. In the conventional VMAT plan, the EUD quartile range for the left hippocampus was 9.315 Gy – 11.325 Gy, and for the right hippocampus was 8.88 Gy – 11.01 Gy. In the HS-VMAT plan, the EUD quartile range for the left hippocampus was 6.6975 Gy – 9.975 Gy, and for the right hippocampus was 6.57 Gy – 9.65 Gy, as shown in **Figure 1**. The HS-VMAT plan can reduce the radiation dose to the left and right hippocampus, thereby lowering the probability of complications in the hippocampal tissue.

**Table 5:** Comparison of EUD values of the left and right hippocampus in the two groups of patients.

Structure	Conventional group	Hippocampus-sparing group	p-value
Left hippocampal EUD	10.055 ± 2.029	8.228 ± 2.888	**0.006**
Right hippocampal EUD	10.059 ± 1.448	8.248 ± 2.243	**<0.001**

Data are expressed as mean ± standard deviation.

P-values < 0.05 are considered statistically significant and are highlighted in bold.

## 4 Discussion

In the present study, consistent with the findings of previous research (7, 8, 16–20) as summarized in **Table 6**, the Hippocampus-Sparing VMAT (HS-VMAT) technique significantly reduced the radiation dose to the bilateral hippocampi compared to conventional VMAT. The HS-VMAT plans achieved a comprehensive and significant reduction in clinically critical hippocampal doses. The substantial decrease in the Dmax, with reductions of up to 10.8-15.75 Gy, directly mitigates the risk of focal damage to neural stem cells within the subgranular zone from high-dose “hot spots” (25). Concurrently, the Dmean to the neural stem-cell niche, which includes the hippocampus, is a powerful predictor of cognitive function preservation, as established by the landmark RTOG 0933 clinical trial and subsequent studies (17). Our HS-VMAT plans successfully constrained the hippocampal Dmean to approximately 11.7 Gy, a level well below the tolerance limits cited in multiple studies, indicating that HS-VMAT can effectively limit the overall radiation dose to the entire hippocampal structure, thereby preserving the neural circuits essential for memory formation and recall. The significant reduction in D40% fulfills the core requirements of the QUANTEC (Quantitative Analysis of Normal Tissue Effects in the Clinic) guidelines and related research (26), which emphasize the control of both overall and critical volume doses to maximally preserve memory-related neural functions. This series of improvements in dosimetric parameters suggests that the HS-VMAT technique holds great promise for significantly reducing the risk of long-term neurocognitive impairment in patients. Notably, the Dmin to the hippocampus did not decrease (27). This is not indicative of an optimization failure but rather reflects both the precision of the VMAT algorithm and its inherent physical limitations. The optimization algorithm successfully carved out the intermediate-to-high dose regions from the hippocampus but could not eliminate the unavoidable low-dose scatter from adjacent target volumes. This result illustrates the limitations of current photon-based radiotherapy and highlights the potential advantages of advanced technologies such as proton and heavy-ion therapy in this domain (28). Crucially, this significant sparing of the hippocampus was not achieved at the expense of treatment efficacy. The dose homogeneity and conformity for the core target volume (PGTV) showed no statistically significant differences between the two groups, ensuring that a radical dose was adequately delivered to the highest-risk area. Although a minor, albeit statistically significant, difference was observed in the homogeneity of the prophylactic irradiation volume (PTV2), this is considered an acceptable dosimetric trade-off for the substantial neuroprotective benefits gained, given that the conformity index remained unchanged. The clinical impact of this subtle change is likely to be negligible. This study confirms that HS-VMAT is a highly balanced optimization strategy. It not only reduces multiple clinically relevant hippocampal dose parameters (Dmax, Dmean, D40%) but also clearly defines its physical limitations (Dmin). Most importantly, it strictly maintains the dosimetric integrity of the core target volume. This body of evidence strongly supports the consideration of HS-VMAT as a superior standard of care for patients with locally advanced nasopharyngeal carcinoma.

**Table 6:** Previous literature studies have summarized the radiation dose received by 40% of the hippocampal volume under the conventional and hippocampus-preserving plans.

Study	Number of patients	Tumor stages	Radiotherapy plans	Conventional VMAT D40%(Gy)	HS-VMAT D40%(Gy)	p-value
Gu, 2017 (21)	11	T3-4	VMAT	13.8	6.4	0.001
Dunlop 2015 (22)	8	T1-4	IMRT/VMAT	23.5	8.6	0.001
Han, 2014 (23)	8	T3-4	IMRT	27.1	13.8	<0.01
Shen, 2020 (24)	52	NA	IMRT	27.2	14.3	<0.001
Peternel 2024 (15)	12	T3-4	VMAT	22.8	13.6	0.002

By constructing a NTCP model and calculating the predicted complication probability for the left and right hippocampi, this study further biologically confirmed that HS-VMAT significantly lowers the predicted probability of radiation-induced complications in the left and right hippocampi compared to conventional VMAT. This finding theoretically supports the advantage of this technique in mitigating radiotherapy-related hippocampal damage. Notably, unlike some studies that treat the hippocampus as a single organ, this study, based on the potentially different roles of the left and right hippocampi in cognitive function, contoured and analyzed the left and right hippocampi as independent OARs. This approach made our study more targeted and helped to reveal potentially different radiosensitivity or protection effects in the two hippocampi. The relatively large sample size also enhanced the reliability and persuasiveness of the study’s statistical analysis results.

However, this study is not without limitations that must be acknowledged. First and foremost, this is a purely dosimetric study. While our findings demonstrate the significant potential of HS-VMAT for neuroprotection by reducing hippocampal doses, we did not assess actual clinical neurocognitive outcomes. Therefore, the presumed clinical benefits, though supported by a large body of literature, remain theoretical within the context of this study. Future prospective trials that correlate these dosimetric advantages with longitudinal neurocognitive function testing are essential to ultimately validate our findings. Second, this was a retrospective study conducted at a single institution with a limited sample size. This may introduce selection bias and limit the generalizability of our results to broader patient populations or different treatment centers. Third, our dosimetric analysis revealed nuances that warrant further investigation. For instance, while the Dmax, Dmean, and D40% of the hippocampi were significantly reduced, the Dmin remained unchanged. We hypothesize that this is due to the low-dose bath from scatter radiation from adjacent target volumes, representing a physical limitation of current photon-based therapy. Similarly, a statistically significant, albeit numerically small, decrease in dose homogeneity was observed in the PTV2. Although we believe this minor dosimetric trade-off is acceptable for the substantial hippocampal sparing achieved, its long-term impact on tumor control probability (TCP) is unknown and requires further investigation. Consequently, the conclusions of this study should be interpreted with caution to reflect the exploratory nature of its dosimetric findings. Our results provide strong support for the feasibility and potential of HS-VMAT, but they cannot definitively prove its clinical superiority over conventional VMAT.

Looking forward, the path to optimizing neurocognitive preservation is multifaceted. Future research should not only focus on prospective clinical validation but also explore the integration of advanced technologies. This includes utilizing artificial intelligence for automated, individualized treatment planning that can better balance target coverage and OAR sparing, as well as exploring the potential of proton therapy to overcome the physical limitations of photon-based Dmin reduction. Furthermore, a comprehensive understanding of post-radiotherapy cognitive decline requires a multi-factorial approach, considering other potential contributing factors such as radiation-induced vasculopathy and endocrinopathies.

## 5 Conclusion

This dosimetric comparative analysis was conducted to explore the potential of HS-VMAT for patients with locally advanced nasopharyngeal carcinoma. The results demonstrate that, compared to conventional VMAT, the HS-VMAT technique can significantly reduce key hippocampal dose parameters (including Dmax, Dmean, and D40%) while maintaining adequate target coverage and effective protection of other organs at risk. These dosimetric advantages suggest that HS-VMAT may be an effective optimization strategy for mitigating radiation-induced hippocampal injury and holds promise for reducing the risk of associated neurocognitive dysfunction. However, the ultimate clinical value of this technique must be confirmed by future prospective studies, particularly clinical trials that include formal neurocognitive assessments.

## Data Availability

The raw data supporting the conclusions of this article will be made available by the authors, without undue reservation.
